# Acupuncture therapy for Alzheimer's disease: A protocol for an overview of systematic reviews

**DOI:** 10.1097/MD.0000000000020244

**Published:** 2020-05-22

**Authors:** Liaoyao Wang, Jia Xu, Yijun Zhan, Jian Pei

**Affiliations:** a Department of Acupuncture; b Special Wards, Longhua Hospital, Shanghai University of Traditional Chinese Medicine, Shanghai, China.

**Keywords:** acupuncture, Alzheimer's disease, AMSTAR 2, GRADE, overview, PRISMA

## Abstract

**Background:**

Alzheimer's disease (AD) is the fifth-leading cause of death in people more than 65 years old. Acupuncture therapy has been traditionally used to treat various kinds of health problems including AD. This protocol aims to summarize the available evidence from current systematic reviews (SRs) for the efficacy of acupuncture therapy for AD.

**Methods:**

Seven databases will be searched: PubMed, Cochrane Library, Embase, China National Knowledge Infrastructure (CNKI), Wanfang Data, Chongqing VIP (CQVIP), and Chinese Biomedical Literature Database (CBM). SRs/meta-analyses (MAs) of acupuncture therapy for AD which were reported in Chinese or English will be included. Study selection, data extraction, and assessment of the study quality will be performed independently by 2 or more reviewers. And the methodological quality, report quality and evidence quality will be evaluated by Assessment of Multiple Systematic Reviews-2 (AMSTAR-2) tool, Preferred Reporting Items for Systematic Reviews and Meta Analyses Statement (PRISMA) checklist and Grading of Recommendations Assessment, Development and Evaluation (GRADE) system, respectively.

**Results:**

The article in this overview will be submitted for publication in a peer-reviewed journal.

**Conclusion:**

We expect to compile evidence from multiple systematic reviews of acupuncture therapy in AD patients in an accessible and useful document.

Registration number: INPLASY202040035.

## Abstract 2

Supplemental Digital Content is available in the text

## 1 Introduction

Dementia is a common global public health problem. There are approximately 47 million people suffered from dementia worldwide and this number is expected to increase to 131 million by 2050.^[1]^ Alzheimer's disease (AD), a neurodegenerative disease, is the main cause of dementia,^[2]^ which accounts for 50% to 75%^[3]^ with extremely harm and characterized by gradual progress of loss of memory, inability to learn new information, cognitive deterioration, mental symptoms, behavioral abnormalities.

In the United States, the prevalence of dementia is 15% in people older than 68 years^[1]^ and about 5.8 million people suffer from AD, which is the fifth-leading cause of death among people older than 65 years.^[1,4,5]^ In China, there are 9.2 million dementia patients of which 62.5% are caused by AD.^[6]^ And AD has become the 14th leading cause of death in Chinese people.^[7]^ It was estimated that China would have over 20 million AD patients in 2050.^[8]^ The disability rate of AD is high, the patients with AD will loss independent living ability, which has caused great burden to family members, nursing staff and the whole society.^[9]^ The annual cost of healthcare related to AD is estimated at nearly $500 billion.^[10]^ Moreover, AD patients often concomitantly have other diseases which may aggravate AD progression and symptoms.^[11]^

Current treatment pharmacologic therapy for AD mainly includes cholinesterase inhibitors and memantine.^[12]^ The cholinesterase inhibitors donepezil, rivastigmine, and galantamine are recommended therapy for patients with mild, moderate, or severe AD. Memantine is approved for use in patients with moderate to severe AD.^[13]^ Besides, Huperzine A is a well-tolerated drug that could significantly improve cognitive performance in patients with AD.^[14]^ And Chinese herbal medicines provide a useful alternative and additive treatment for AD.^[15,16]^

Nonpharmacologic therapy including physical exercise, cognitive stimulation programs, art therapy, and memory training show potential benefit in treatment for AD patients.^[1,17]^ These nonpharmacologic therapies are often used to maintain or improve cognitive function, the ability to perform activities of daily living, or overall quality of life. And the behavioral symptoms such as depression, apathy, wandering, sleep disturbances, agitation, and aggression may be reduced.^[5]^

However, in the present there are still no treatments available to slow down or stop the damage and destruction of neurons^[5]^ and disease progression effectively,^[11]^ although the current medications used to treat AD are able to alleviate the symptoms.

Acupuncture is a unique nonpharmacologic therapy which protects neurons from degeneration and promotes axonal regeneration in neurodegenerative diseases such as AD.^[18]^ In recent years, increasing evidence show that acupuncture may be an effective and safe way to treat AD.^[19–22]^ However, 1 SR showed that the existing evidence is not able to prove the efficacy of acupuncture therapy for AD.^[23]^ In another SR, evidence on the efficacy of acupuncture in improving cognitive function in patients with AD was insufficient.^[24]^ There are significant differences among the results, which is not conducive to the evaluation and use of clinicians. Therefore, we conduct an overview of systematic reviews (SRs) and meta-analyses (MAs) of acupuncture therapy for AD.

## 2 Objectives

The objectives are as following:

(1) Comprehensively assess the quality including methodological quality, report quality, and evidence quality of SR of acupuncture for AD, and find out what can be improved.
(2) To summarize the available evidence from current SRs for the effectiveness and safety of acupuncture for AD and to provide reference for clinical practice of acupuncture therapy for AD.

## 3 Methods

This protocol of overview will be performed according to “preferred reporting items for overview of systematic reviews” (PRIO-harms).^[25]^ This overview has been registered on the International Platform of Registered Systematic Review and Meta-analysis Protocols (INPLASY), registration number: INPLASY202040035, DOI number: 10.37766/inplasy2020.4.0035 (https://inplasy.com/inplasy-2020-4-0035/).

### 3.1 Criteria for considering reviews for inclusion

Published SRs which were reported in Chinese or English, will be considered for inclusion in this overview. Protocols, meeting abstracts, and other reviews will be excluded.

#### 3.1.1 Types of participants

We will include patients with AD. No restrictions on age and gender.

#### 3.1.2 Types of interventions

Acupuncture or acupuncture plus drug. Manual acupuncture or electropuncture is available and there is no limitation of the type of drugs.

#### 3.1.3 Types of comparisons

Comparisons will include the drugs or no treatment.

#### 3.1.4 Types of outcomes

(1) *Mini-mental state examination (MMSE)*: MMSE was developed more than 4 decades ago.^[1]^ MMSE is a simplified, scored form of the cognitive mental status examination. It is “mini”, but the realm of cognitive is thorough. And it requires only a few minutes to administer, therefore it is practical to use routinely.^[26]^
(2) *Activities of daily living (ADL)*: ADL measurement method was proposed by Katz, which was developed to study results of treatment and prognosis in the elderly and chronically ill. It offers a means of making quantitative assessments.^[27]^ The 6 basic activities in the ADL consist of eating, dressing, indoor mobility, bathing, using the toilet, and continence.^[28]^
(3) *Alzheimer's disease assessment scale-cognitive section (ADAS-cog)*: The ADAS is a rating instrument which was designed specifically to evaluate the severity of cognitive and noncognitive behavioral dysfunctions characteristic of persons with AD.^[29]^

#### 3.1.5 Types of studies

We will include SR of randomized control trials (RCTs) which evaluate the effect and safety of acupuncture therapy for AD.

### 3.2 Data collection

#### 3.2.1 Search methods for identification of reviews

A comprehensive and exhaustive search strategy will be run. The following databases: PubMed, Embase, Cochrane Library, China National Knowledge Infrastructure (CNKI), Chinese Biomedical Literature Database (CBM), WANFANG DATA, VIP will be searched using the keywords (“Alzheimer Disease” OR “Senile Dementia”) AND (“Acupuncture∗” OR “Electroacupuncture” OR “scalp needle”) AND (“Meta analys∗” OR “Systematic review∗”). The Search strategy was given in Supplemental Digital Content (Appendix 1).

#### 3.2.2 Selection of reviews

Search results will be exported into Endnote X8. Duplications will be removed, and then the irrelevant literatures will be excluded after screening titles and abstracts. According to inclusion and exclusion criteria, reviews that may be eligible will be read the full text and those meet the criteria will be selected. Two of the review authors (Liaoyao Wang and Jia Xu) will independently assess reviews, and any disagreements will be resolved by a third review author (Jian Pei).

#### 3.2.3 Data extraction and management

Data extraction form will be designed including detailed information of each review. Data will be extracted independently by 2 overview authors (Jia Xu, Yijun Zhan) using an Excel spreadsheet. We will resolve any discrepancies by discussing with a third review author. The overview will contain a characteristics of included reviews table. This table will include the following information: first author, publication year, database, number of literatures, sample size, interventions, outcomes, quality assessment tools, etc (Table 1).

**Table 1:**
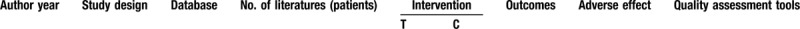
Characteristics of included reviews.

#### 3.2.4 Critical appraisal of included reviews

The following evaluation processes were performed independently by 2 authors. If the opinions are inconsistent, all the researchers will discuss on the meeting until reach a consensus. We will calculate the rate of agreement between the 2 reviewers. Agreement will be measured using the Kappa statistic.^[30]^

### 3.3 Data synthesis

We will provide a narrative description of the findings of the included SR. Tables will be produced to detail the included studies and their outcomes. In addition, we will synthesis these reviews and provide pooled treatment effects for all SRs which include the following outcomes: MMSE, ADL, ADAS-cog score. For each of our outcomes we will perform a sub-group analysis comparing acupuncture vs drugs, acupuncture combined with drugs vs drugs. If necessary, the results will combine in a meta-analysis, the statistical analyses were conducted using the RevMan5.3 software. The summary effect size was estimated by using mean difference (MD) with 95% confidence intervals (CI) for continuous outcomes.

### 3.4 Evaluation of the quality of the included reviews

#### 3.4.1 Assessment of methodological quality of included reviews

We will rate the methodological quality of each SR using the AMSTAR 2 tool.^[31]^ The AMSTAR 2 tool contains 16 domains, 7 of which are critical domains (2, 4, 7, 9, 11, 13, 15). According to evaluation criteria, each systematic review will be categorized into “high confidence” (if there is no critical weakness and no or only 1 non-critical weakness); “moderate confidence” (if there is more than 1 non-critical weakness with no critical weakness); “low confidence” (if there is 1 critical weakness with or without non-critical weaknesses); and “critically low confidence” (if there is more than 1 critical weakness with or without non-critical weaknesses).

#### 3.4.2 Report quality of included reviews

We will also use Preferred Reporting Items for Systematic Reviews and Meta Analyses Statement (PRISMA) checklist^[32]^ to assess the report quality of the included reviews by tabulating whether the following items have been adequately addressed. It is 1 point for the content of the item, 0.5 point for the part, and 0 point if the systematic review does not match the entry. The total score ≥22 is “high quality”, 11 to 21 is classified as “moderate quality”, and ≤10 is classified as “low quality” (Table 2).

**Table 2:**
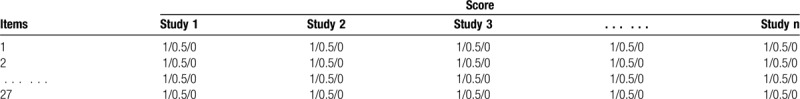
PRISMA checklist item.

#### 3.4.3 Quality of evidence in included reviews

We will describe the quality of the evidence using the Grade of Recommendation, Assessment, Development, and Evaluation (GRADE) system.^[33]^ The evidence for each of the selected clinical outcomes in the table which will be filled with the summary of estimated-risk and 95% confidence intervals (Table 3). The quality of evidence of each outcome will be ranged from high, moderate to low, and very low. The evidence can be downgraded from “high quality” by 1 level for serious (or by 2 levels for very serious) limitations, depending on assessments for study limitations, inconsistency of results, indirectness of evidence, imprecision of effect estimates, and potential publication bias.

**Table 3:**
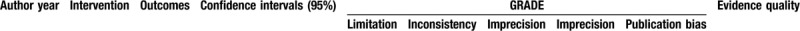
GRADE quality rating.

## Author contributions

Liaoyao Wang conceived the idea of research and developed the first draft of the manuscript. Jia Xu developed the draft search strategy plan. Yijun Zhan and Jia Xu revised several versions of the manuscript. Jian Pei is the guarantor and he approved the final version.

**Conceptualization:** Liaoyao Wang, Jian Pei.

**Data curation:** Liaoyao Wang, Jia Xu.

**Formal analysis:** Jia Xu, Yijun Zhan.

**Investigation:** Jia Xu, Yijun Zhan.

**Methodology:** Liaoyao Wang, Jia Xu, Yijun Zhan, Jian Pei.

**Project administration:** Liaoyao Wang, Jia Xu, Yijun Zhan, Jian Pei.

**Supervision:** Jia Xu, Jian Pei.

**Validation:** Jian Pei.

**Visualization:** Liaoyao Wang, Jia Xu, Yijun Zhan.

**Writing – original draft:** Liaoyao Wang.

**Writing – review & editing:** Jian Pei, Jia Xu.

## Supplementary Material

Supplemental Digital Content
