# Supplementary material for: Acupuncture therapy for Alzheimer's disease: A protocol for an overview of systematic reviews
Source: Medicine (Baltimore). 2020 May 22;99(21):e20244. doi: 10.1097/MD.0000000000020244 (PMC7249950; doi:10.1097/MD.0000000000020244)
Supplement: Supplemental Digital Content [file medi-99-e20244-s001.doc]

**Appendix 1 Search strategy**

(1) PubMed

| **NO.** | **Search strategy** | **items** |
| --- | --- | --- |
| #1 | "Alzheimer Disease"[Mesh] |  |
| #2 | (Alzheimer Disease[Title/Abstract]) OR Senile Dementia[Title/Abstract] |  |
| #3 | #1OR#2 |  |
| #4 | ("acupuncture"[Mesh]) OR ("Acupuncture Therapy"[Mesh]) |  |
| #5 | ((Acupuncture*[Title/Abstract]) OR Electroacupuncture[Title/Abstract]) OR scalp needle[Title/Abstract] |  |
| #6 | #4 OR #5 |  |
| #7 | "Meta-Analysis" [Publication Type] OR "Meta-Analysis as Topic"[Mesh] |  |
| #8 | (Meta analys*[Title/Abstract]) OR Systematic review*[Title/Abstract] |  |
| #9 | #7 OR #8 |  |
| #10 | #3 AND #6 AND #9 |  |

(2) Embase

| **NO.** | **Search strategy** | **items** |
| --- | --- | --- |
| #1 | 'alzheimer disease'/exp |  |
| #2 | 'alzheimer disease':ab,ti OR 'senile dementia':ab,ti |  |
| #3 | #1 OR #2 |  |
| #4 | 'acupuncture'/exp |  |
| #5 | acupuncture*:ab,ti OR electroacupuncture:ab,ti OR 'scalp needle':ab,ti |  |
| #6 | #4 OR #5 |  |
| #7 | 'meta analysis'/exp |  |
| #8 | 'meta analys*':ab,ti OR 'systematic review*':ab,ti |  |
| #9 | #7 OR #8 |  |
| #10 | #3 OR #6 OR #9 |  |

(3) Cochrane Library

| **NO.** | **Search strategy** | **items** |
| --- | --- | --- |
| #1 | MeSH descriptor: [Alzheimer Disease] explode all trees |  |
| #2 | ("Alzheimer Disease"):ti,ab,kw AND ("Senile Dementia"):ti,ab,kw |  |
| #3 | #1 OR #2 |  |
| #4 | MeSH descriptor: [Acupuncture] explode all trees |  |
| #5 | (Acupuncture*):ti,ab,kw OR (Electroacupuncture):ti,ab,kw OR ("scalp needle"):ti,ab,kw |  |
| #6 | #4 OR #5 |  |
| #7 | MeSH descriptor: [Meta-Analysis] explode all trees |  |
| #8 | ("Meta analys*"):ti,ab,kw OR ("Systematic review*"):ti,ab,kw |  |
| #9 | #7 OR #8 |  |
| #10 | #3 AND #6 AND #9 |  |

(4) Chinese databases

| **Databases** | **Search strategy** | **items** |
| --- | --- | --- |
| CNKI | (阿尔茨海默 OR 老年性痴呆OR AD) AND (针灸OR针刺OR电针OR头针) AND (系统评价OR系统综述OR荟萃分析OR meta分析OR元分析) |  |
| WANFANG DATA |  |
| VIP |  |
| CBM |  |
